# Vestibular Hearing and Speech Processing

**DOI:** 10.5402/2012/850629

**Published:** 2012-02-14

**Authors:** Seyede Faranak Emami, Akram Pourbakht, Kianoush Sheykholeslami, Mohammad Kamali, Fatholah Behnoud, Ahmad Daneshi

**Affiliations:** ^1^Department of Audiology, School of Rehabilitation, Tehran University of Medical Sciences, Tehran 16997-387, Iran; ^2^American Academy of Otolaryngology-Head and Neck Surgery Rockford, OSF Saint Anthony Medical Center, IL, USA; ^3^Department of Statistic, School of Rehabilitation, Tehran University of Medical Sciences, Tehran, Iran; ^4^Department of Otorhinolaryngology/Head and Neck Surgery, School of Medicine, Hamadan University of Medical Sciences, Hamadan 16657-696, Iran; ^5^ENT-Head and Neck Research Center, Hazrat Rasoul Akram Hospital, Tehran University of Medical Sciences, Tehran 14455-364, Iran

## Abstract

Vestibular hearing in human is evoked as a result of the auditory sensitivity of the saccule to low-frequency high-intensity tone. The objective was to investigate the relationship between vestibular hearing using cervical vestibular-evoked myogenic potentials (cVEMPs) and speech processing via word recognition scores in white noise (WRSs in wn). Intervention comprised of audiologic examinations, cVEMPs, and WRS in wn. All healthy subjects had detectable cVEMPs (safe vestibular hearing). WRSs in wn were obtained for them (66.9 ± 9.3% in the right ears and 67.5 ± 11.8% in the left ears). Dizzy patients in the affected ears, had the cVEMPs abnormalities (insecure vestibular hearing) and decreased the WRS in wn (51.4 ± 3.8% in the right ears and 52.2 ± 3.5% in the left ears). The comparison of the cVEMPs between the subjects revealed significant differences (*P* < 0.05). Therefore, the vestibular hearing can improve the speech processing in the competing noisy conditions.

## 1. Introduction

cVEMPs provide a means of assessing otolith function. Stimulation of the vestibular system with air-conducted sound activates predominantly saccular afferents, Wu and Young [1], Curthoys et al. [2], and Wang et al. [3]. The auditory sensitivity of the saccule (vestibular hearing) has been demonstrated in amphibians, birds, mammals, and among human, Curthoys et al. [4], Eatock and Lysakowski [5], and Zhou et al. [6]. The frequencies between 50−800 Hz above 90 dB SPL evoke vestibular hearing Todd et al. [7], Sheykholeslami et al. [8], and Sheykholeslami and Kaga [9]. The range of vestibular hearing happens to coincide with the range of our voice pitch, Todd et al. [7], which varies considerably among men (F0 =*∼*100 Hz), women (F0=*∼*200 Hz), and children (F0 = up to 400 Hz), Abrams and Kraus [10]. Also, there is a vestibular pathway to middle ear muscles. The saccular afferents may also give rise to a response in stapedius. This evidence suggests that the saccule retains an ability to trigger acoustic reflexes of certain muscles in man, which may serve an “antimasking” function of low frequency on high-frequency-tones, Rosengren et al. [11] and Sheykholeslami and Kaga [9]. The olivocochlear efferent system during stapedial reflex acts to extend dynamic range; this feedback system can be expected to exert a greater effect on the representation of speech to loud sounds in human. It can improve the representation of spectral shape and speech perception at high stimulus levels, Todd et al. [7], Sheykholeslami and Kaga [9], and Curthoys et al. [2].

There is anatomical evidence of a projection from the saccular nerve into the cochlear nucleus, Todd et al. [7], Sheykholeslami et al. [8]. Some of the primary vestibular afferent nerves send projections to various auditory fields on the cortex recorded acoustically evoked neural activity from the brainstem and auditory cortex of guinea pigs, Sheykholeslami and Kaga [9]. The areas of the brain that activate from vestibular hearing consist of the primary visual cortex, the precuneus, the precentral gyrus, the medial temporal gyrus, and the superior temporal gyrus, McNerney et al. [12].

The data available for hearing impaired subjects show some evidence of changes in the pattern of discriminability for tones above vestibular hearing threshold, Todd et al. [7] and Sheykholeslami and Kaga [9]. It is a fact that intensities in the vocal tract can be very high, the sound pressure to be as high as 130 dBSPL (between 94 and 106 dBSPL). Thus, the vestibular hearing may be obtained to an individual's own production or when there are large groups of individuals vocalizing together, such as speech sounds that are presented in background noise in a choir or a crowd at a concert, Todd et al. [7].

Therefore, the objective was to investigate the relationship between vestibular hearing using cervical vestibular-evoked myogenic potentials (cvemps) and speech processing via word recognition scores in white noise (WRSs in wn).

## 2. Materials and Methods

### 2.1. Participants

The study involved twenty healthy controls, which consisted of audiology students and hospital staff (10 females and 10 males, mean age 30 years and range 20–39 years). The case group was twenty two dizzy patients selected from subjects who presented with the complaint of disequilibrium (14 females and 8 males, mean age 32 years and range 20–39 years), which diagnosed with benign paroxysmal positional vertigo, migraineur, and psychogenic causes. The dizzy patients were consecutive subjects who presented to the Audiology Department of Tehran University of Medical Sciences (all 42 persons were volunteers). All the subjects received detailed information about the study and the testing that would be involved. Informed consent was obtained from each individual, and the study was approved by Tehran University of Medical Sciences. The exclusion criteria was the history of ear infections and middle ear diseases, which could interfere with cVEMPs, DPOAE, and IA measurements.

### 2.2. Assessments

In our research, total of eighty-four ears were evaluated, which had normal otoscopy findings. Testing was performed bilaterally and intervention comprised of *pure tone audiometry *(PTA),* impedance acoustic *(IA), *distortion product otoacoustic emissions *(DPOAEs)*, click-evoked or fast component auditory brainstem response* (ABR),* videonystagmography *(VNG), cVEMPs, and WRSs in wn using the standard devices.

Also, during the process, we ensured that the persons were attended to their task. The social status and sex were not taken into consideration. All of tests performed on same day. In each step of evaluation, when the procedure was completed for the one test, subjects were given a short break and the whole procedure repeated for another. A handedness questionnaire was also administered. All the subjects were right-handed, and they were native speakers of persian language (with uniLinguistic abilities).

#### 2.2.1. Pure Tone Audiometry (PTA)

PTA thresholds in the normal range (−10 to 15 dBHL) were obtained from each person over the frequency range of 250–8000 Hz, Harrel [13].

#### 2.2.2. Impedance Acoustic (IA)

For the impedance *acoustic*, middle-ear pressure between the limits of ±  50 mm H_2_O was evaluated. The values that were out of this limit were omitted from the analyses, Fowllff and Shanks [14]. 

#### 2.2.3. Distortion Product Otoacoustic Emissions (DPOAEs)

DPOAE was measured in white noise (WN). The *f*1/*f*2 ratio was fixed at 1.22, and stimulus levels were held constant at  *L*1 = 65 dBSPL and *L*2 = 55 dBSPL. The 2*f*1 − *f*2 DPOAE amplitudes were recorded at frequencies (*f*2) 1.0, 1.5, 2.0, 3.0, 4.0, 6.0, and 8.0 kHz. The WN was delivered at 30 dBHL. The DPOAE amplitudes were considered significant when they were at least 3 dB above the noise floor. The averaged values less than 3 dB were omitted from the analyses, Mukari and Mamat [15].

#### 2.2.4. Fast Component Auditory Brainstem Response (fABR)

Responses were recorded from the subjects in the supine position, with electrodes placed at the high forehead and on each mastoid. The electrode contralateral to the ear of stimulation served as ground. The impedance between any two electrodes was below 5 k ohms. Filter roll-off rate was 6 dB per octave (bandpass filtered from 30 Hz to 3000 Hz). The averaged time window was 20 msec, and 2000 stimulus presentations were incorporated into each averaged response. Each trace was replicated. The click stimulation was delivered monaurally with contralateral masking (click = 80 dBSPL; sound pressure level, noise = 50 dBSPL) to ER-3A insert earphones with alternating polarity at a rate of 2l/sec, Gorga et al. [16]. We considered the *f*ABR to be abnormal when peaks III and/or V were absent or when the peak to peak I-V exceeded the normal limits of our laboratory (4.40 ms for females, 4.58 ms for males). The averaged values that were out of the normal limit were omitted from the analyses.

#### 2.2.5. VideoNystagmoGraphy (VNG)

VNG was conducted to eliminate the possibility of any additional vestibular pathology. The battery of VNG tests included assessment of the central vestibular and vestibulocular systems with evaluation of gaze. VNG was consisted of *Smooth Pursuit test* (the trajectory of the target was typically predictable and the frequency of movement less than approximately 1.2 Hz). *Saccade test* (the difference between the position of the target on the retina and the desired position on the fovea calculated as retinal slip). *Optokinetic test* (jerk nystagmus eye movements created by repeated objects moving across the subject's visual field and filling at least 80% of the visual field). *Gaze Fixation and Spontaneous Nystagmus *(the Jerk nystagmus was the principal abnormality interest in most situations). *Positive Hallpike responses*, which analyzed by torsional nystagmus. The use of positional nystagmus was an indicator of peripheral system lesion. Our criteria for clinical significance were based on persistent nystagmus in four or more of the eight to eleven positions. *Caloric stimulation,* the absence of a response to warm and cool air was not an indication of complete lack of function. Caloric test and the head impulse test (HIT) were used to define semicircular canals function, Shepard [17], Barin [18], and Cha [19].

#### 2.2.6. Cervical Vestibular-Evoked Myogenic Potentials (cVEMPs)

During cVEMPs recording, patients were instructed to turn and hold their heads as far as possible toward the side contralateral to the stimulated ear. Moreover, one examiner by the finger force on their back head has kept the corrected position. The active electrode was placed over the middle portion of the ipsilateral SCM muscle body as this location appears to generate the most reliable and consistent responses. The reference and the ground electrodes were placed over the upper sternum and on the midline forehead, respectively, Curthoys et al. [4]. Auditory stimuli consisted of tone burst (500 Hz, 120 dB peak SPL), rise/fall time = 1 ms, plateau = 2 ms), presented to the ear ipsilateral to the contracted SCM muscle, bandpass-filtered (20 Hz to 2 kHz), and a grand average of the 200 responses calculated by a standard evoked potential recorder. The latencies, amplitudes, and peak-to-peak amplitudes of these waves were calculated and recorded. For each subject, the cVEMPs asymmetry ratio (evoked potential ratio) was calculated according to the formula of Murofushi et al.: 100[(*A*_*n*_ − *A*_*d*_)/(*A*_*n*_ + *A*_*d*_)], where *A*_*n*_ = p13-n23 (the peak-to-peak amplitude in the normal ear) and *A*_*d*_  = p13-n23 (the peak-to-peak amplitude in the affected ear), Rosengren et al. [11], and Murofushi et al. [20].

In bilateral case, cVEMPs asymmetry ratio is not calculated. In the control group, this ratio was calculated using the peak-to-peak amplitudes for the right ear and the left ear, respectively. The cVEMPs results for the control group were used as normative data. The normative values for latency and cVEMPs asymmetry ratio were calculated as mean ± two standard deviations, Murofushi et al. [20]. Latencies longer than the calculated upper limit were interpreted as abnormal. Any cVEMPs asymmetry ratio above the calculated upper limit (mean + two standard deviations) was considered to reflect depressed response on the side with lower amplitude findings and was interpreted as abnormal. Absence of a meaningful waveform with p13 and n23 (no response) was also considered as an abnormal finding. 

#### 2.2.7. Speech Evaluations

The speech assessments consisted of *speech reception threshold *(SRT)*, most comfortable level *(MCL)*, uncomfortable level *(UCL), WRS in quiet, and WRS in wn. SRTs were assessed using the standard two-syllable words list (developed in Audiology Department of Tehran University of Medical Sciences). MCL and UCL were determined in the normal range. WRS in quiet was tested using the standard monosyllable phonetically balanced words list. The words were presented by one female (monitoring of live voice), who was the native Persian and had no the dialect. She did not know about the case or the control subjects, and testing was randomized (blind). All subjects (case and control) had normal scores, 96–100%, Brandy [21]. Indeed, there is no noticeable systematic differences in consonant scores, voicing scores, and consonant confusions for male and female talker utterances, Lovitt and Allen [22]. 

#### 2.2.8. Word Recognition Scores in White Noise (WRS in wn)

Regarding the phonological properties of persian language, which have 6 vowels (/i/, /e/, /æ/, /a/, /o/, /u/), and depending on frequency characteristics of each vowel (F1) (see Table 1), Wikipedia (Acoustic Phonetics Formants), we created two different monosyllabic words lists. They were common at low-error set of consonants (/v/, /m/, /n/). This consonants have least affection from noise spectrum [22]. The first list was combined with low-frequency vowels (/u/, /i/) and the second list with high-frequency vowels (/æ/, /*ɑ*/). Each list has been made of twenty five monosyllabic words and presented at 10 dB signal to noise ratio (signal = 95 dBHL and white noise = 85 dBHL) to subjects' ipsilateral test ear at the same time. 

### 2.3. Data Analyses

Data were analyzed by unpaired Student's *t*-tests and one-way ANOVA for continuous variables and *χ* ^2^-tests for categorical variables. A *P* value of <  0.05 was considered to indicate statistical significance.

## 3. Result

### 3.1. VideoNystagmoGraphy (VNG)

The dizzy patients presented with a total of forty-four ears (%52.2 affected ears or 23 presented with peripheral vestibulopathic, and %47.2 unaffected ears or 21 contralateral normal ears). The affected ears consisted of benign paroxysmal positional vertigo (11 ears; 25%), migraineurs (5 ears; %11.4), vestibular neuritis (2 ears; %4.4), and psychogenic causes (5 women, 5 ears; %11.4), which had the symptom of a uniattacked of sudden vertigo during few hours after *divorce, strife, *and *death of father*.

The diagnosis of patients with BPPV found severe vertigo that lasts for seconds and is provoked by head movements and results of typical nystagmus (torsional up beating nystagmus with latency and fatigue lasting less than 1 min) and subjective vertigo in the Dix-Hallpike, Barin [18] and Cha [19].

The diagnosis of patients with vestibular neuritis obtained on a history of severe continuous vertigo, they had a feeling of nausea or vomiting. VideoNystagmoGraphy showed a caloric paresis and abnormal head impulse test in the affected ear, Fujimoto et al. [23] and Cha [19].

In migraineurs, vestibular dysfunctions were connected with nystagmus and with episodic vertigo, or a variety of combinations of headache/vertigo. A number of patients reported that their symptoms were worse with achieved head status, but this is not confused with BPPV, since patients with migrainous vertigo were nauseated or phonophobic during attacks, Cha [19].

The diagnosis of psychogenic causes is obtained in the General Health Questionnaire (GHQ), as assigned by Willmott et al. [24], and the positive head thrust test + hypo-caloric responses (abnormalities in VNG), Teggi et al. [25].

In dizzy patients, the direction-fixed nystagmus was interpreted to indicate pathology within the peripheral system. Also, the ocular motor evaluation was normal (which is an index for central vestibular lesions). The other forms of abnormal eye movements were such as pendular nystagmus (sinusoidal, horizontal, repeating eye movements not observed), Shepard [17].

Therefore, twenty-one patients were ipsilesional affected, and one patient with BPPV was affected bilaterally. Thirteen out of twenty-one unilateral cases were affected on the right side and eight on the left side.

### 3.2. Cervical Vestibular-Evoked Myogenic Potentials (cVEMPs)

**Figure 1:**
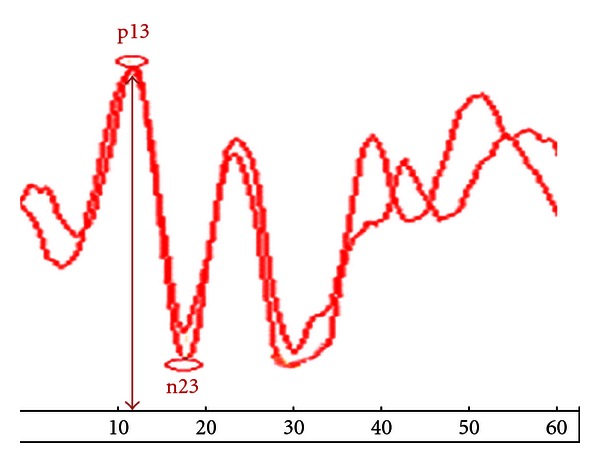
Cervical vestibular-evoked myogenic potentials (cVEMPs) in healthy subject.

Testing of cVEMPs was done in both ears of each control subject (20 right and 20 left ears). The latency and the amplitude values of cVEMPs were detectable in all healthy persons (40 ears with safe vestibular hearing). The mean latency values for p13 and n23 were 12.7 ± 1.0 and 20.1 ± 2.2 ms, respectively (Table 2). Therefore, the upper limits (mean + two standard deviations) for latency at p13 and n23 in our study were 14.7 and 24.5 ms, respectively. The mean peak-to-peak amplitude in the control group was 25.9 ± 23.8 *μ*v. The mean cVEMPs asymmetry ratio was 6.5 ± 10.2%, and the upper limit for this ratio (two standard deviations above the mean) was 26.9%. A recording from a normal subject is given in Figure 1.

The cVEMPs abnormalities (insecure vestibular hearing) were included: both decreased amplitudes and delayed latencies in twelve (1 psychogenic subject, 7 BPPV, 4 migraineurs) and absent responses in eleven (2 vestibular neuritis, 4 psychogenic subjects, 4 BPPV, 1 migraineurs). In the BPPV group, the mean latencies at p13 and n23 were 15.12 ± 1.33 ms and 24.69 ± 1.19 ms, respectively. The mean peak-to-peak amplitude was 24.6 ± 1.4 *μ*v. In the migraineurs, the mean latencies at p13 and n23 were 15.77 ± 1.36 ms and 25.33 ± 0.55 ms, respectively. The mean peak-to-peak amplitude was 23.8 ± 1.9 *μ*v. In the psychogenic subjects, the mean latencies at p13 and n23 were 14.9 ± 1.5 ms and 24.8 ± 1.2 ms, respectively. The mean peak-to-peak amplitude was 25.3 ± 2.1 *μ*v (Table 2).

**Figure 2:**
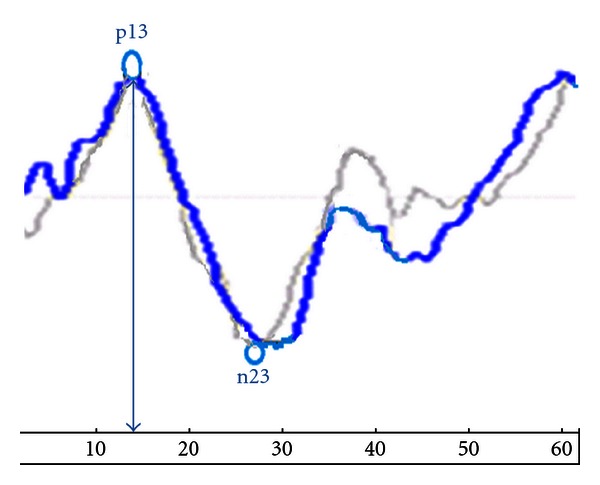
Cervical vestibular-evoked myogenic potentials (cVEMPs) in dizzy patient.

In all dizzy patients, the cVEMPs asymmetry ratio findings indicated depressed response on the side with lower amplitude findings in a single ear only. The mean p13 and n23 latencies in the affected ears were both longer than the respective means in the control group; also, the differences were significant (*P* < 0.05 for both). The mean peak-to-peak amplitude in the affected ears was significantly lower than that in the control group (*P* < 0.05). A recording from a dizzy patient is given in Figure 2.

### 3.3. Word Recognition Scores in White Noise (WRSs in wn)

**Figure 3:**
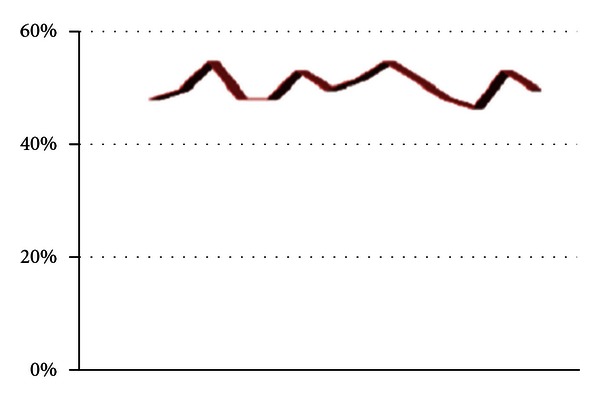
Word recognition scores in white noise (WRSs in wn) at the affected ears of the dizzy patients on the right.

**Figure 4:**
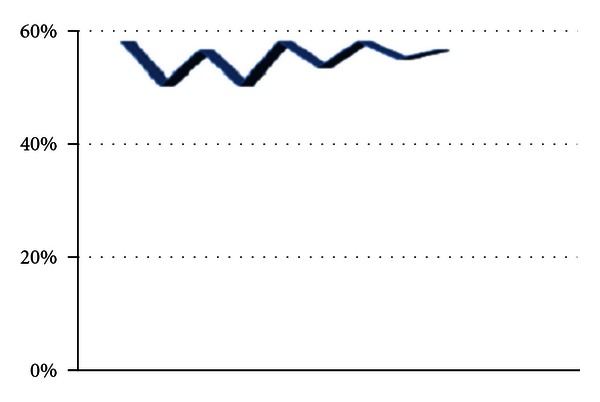
Word recognition scores in white noise (WRSs in wn) at the affected ears of the dizzy patients on the left.

WRS in wn obtained for all healthy subjects (66.9 ± 9.3% in the right ears, 67.5 ± 11.8% in the left ears) and the upper limits (mean + two standard deviations) for the right ears and the left ears was 85.5% and 91.1%, respectively. There were no significant differences (*P* > 0.05) between the healthy subjects and unaffected ears of the dizzy patients, whereas the dizzy patients in affected ears had decreased WRS in wn (51.4 ± 3.8% in the right affected ears, and 52.2 ± 3.5% in the left affected ears). Word recognition scores in white noise (WRSs in wn) at the affected ears of the dizzy patients on the right is given in Figure 3. The upper limits for the right ears and the left ears were 59% and 59.2%, respectively, (Table 3). Also, there were no significant difference (*P* > 0.05) between the affected ears on monosyllabic words lists. However, the recognition rates of vowels heavily depend on the duration of their phonemes [22]. Word recognition scores in white noise (WRSs in wn) at the affected ears of the dizzy patients on the left is given in Figure 4.

**Final Result**

Our main outcome measures were differences in amplitudes, p13 - n23 latencies of the cVEMPs between affected ears (23 ears with insecure vestibular hearing and worse WRS in wn), and unaffected ears (21 ears with safe vestibular hearing and better WRS in wn), respectively. Comparison of the cVEMPs at affected ears versus unaffected ears and the normal persons revealed significant differences (*P* < 0.05). Thus, safe vestibular hearing improved word recognition scores in white noise, and insecure vestibular hearing decreased word recognition scores in white noise.

## 4. Discussion

Better central auditory function is usually associated with better word recognition scores in white noise, Yilmaz et al. [26]. The individuals with normal hearing include words recognition scores in quiet ≥ 90%, and they have the average obtain 50% performance at a signal-to-noise ratio of 2 to 6 dB, Brandy [21]. Also, for adults with normal hearing, the mean of word recognition scores in white noise at 10 dB signal to noise ratio is equal to 67.7%, Meyer et al. [27]. In our study, the total mean of scores at 10 dB signal-to-noise ratio for all subjects was in the normal range. The upper limit of scores was acquired for the healthy persons, whereas the lower limit of scores was obtained for affected ears. One reason for low word recognition scores in white noise may be poor auditory processing, Meyer et al. [27] and Yilmaz et al. [26]. Thus, low-frequency-high-intensity function of vestibular hearing may be useful in auditory processing.

However, the low-frequency cues have very important roles to detect a word in noise. It is important to note that, during listening in silence, auditory neurons respond to both fundamental frequency tones (F0) and nonfundamental frequency tones, whereas under conditions favoring perception of two separate auditory signals (listening in noise), auditory neurons respond only to F0 tones, Fishman and Steinschneider [28], Abrams and Kraus [10]. Thus, low-frequency components of our voice (F0) at overt articulation can induce vestibular hearing, Todd [7, 29], which is effective in speech understanding. Also, the speech understanding depends on accurate perception by the listener, whether in terms of discrimination, identification, recognition, or comprehension, Scott and Sinex [30] and McCreery et al. [31].

The low-frequency components in speech (F0 and F1; first formant), as assigned by Mukari and Mamat [15] which set in vestibular hearing range, Todd [7, 29], conveys the phonetic information and prosodic cues, such as intonation and stress, Scott and Sinex [30]. The brainstem is phase-locked to F0, Abrams and Kraus [10], and the left hemisphere insular is specifically activated when F0 provides lexical information to a native speaker, Scott and Sinex [30]. The brainstem encodes F1 tones, which is critical to vowel perception, May [32] and Scott and Sinex [30]. Also, primary and nonprimary regions of temporal cortex are sensitive to aspects of F1 and F2 that are essential for normal perception, it is spatially mapped in the cortex, Abrams and Kraus [10] and Sekaran and Kruas [33]. Therefore, vestibular hearing can contribute to frequency discrimination of loud tones and can improve speech perception.

Indeed, in a clamor situation, better vestibular hearing can help to sound localization on interaural time difference (ITD), which is effective at low frequencies, Tom and Shigeyuki [34] and May [32]. Extending the representation to large lTD sensitivity may be useful not only for sound localization, but also for gaining information about auditory space and auditory scene analysis, which are the basis of hearing, Tom and Shigeyuki [34]. Because the deficits in auditory scene analysis may partly underlie hearing difficulties. Other aspects of scene analysis do appear to rely on prior learning, attention, and other “top-down” processes that further constrain inferences made by the brain concerning the environmental sound sources giving rise to the vestibule-cochlear hearing, Fishman and Steinschneider [28]. Thus, vestibular hearing may be effective in top-down processing of loud sounds.

In addition, babies do use pitch variations (the range of vestibular hearing) to segment words, Hartley and King [35], and, in tonal languages, adults need pitch variations to understand speech, Abrams and Kraus [10]. So speech perception/production are linked at mapping sounds to articulations, mapping articulations to sounds, and suppressing the neural response to own vocalizations in brain areas associated with the processing of speech. When listening to words in noise, brain areas associated with semantic processing and speech production are recruited and potentially indicate the use of overt strategies by the listeners; for example, using overt articulation (the range of vestibular hearing) to increase perception, Scott and Sinex [30]. Consequently, vestibular hearing may be valuable for speeh processing and perception/production system.

## Figures and Tables

**Table 1:** 

Vowel	[i]	[I]	[e]	[*ε*]	[æ]	[*ɑ* **]**	[*ɔ*]	[o]	[*℧*]	[u]	[Λ]
F1 (HZ)	280	370	405	600	860	830	560	430	400	330	680
F2 (HZ)	2230	2090	2080	1930	1550	1170	820	980	1100	1260	1310

**Table 2:** Mean of the right and left latencies and amplitudes of cVEMPs in healthy persons and dizzy patients.

Subject	Lp13 (Ms)	LN23 (Ms)	INA (*μ*v)
P	14.9 ± 1.5	24.8 ± 1.2	25.3 ± 2.1
BPPV	15.12 ± 1.33	24.69 ± 1.19	24.6 ± 1.4
M	15.77 ± 1.36	25.33 ± 0.55	23.8 ± 1.9
VN	Absent	Absent	Absent
Healthy	12.7 ± 1.0	22.1 ± 2.2	25.9 ± 23.8

BPPV: benign paroxysmal positional vertigo, VN: vestibular neuritis, M: migraineur, P: psychogenic, Lp13: latency P13, LN23: latency N23, INA: interpeak amplitude.

**Table 3:** The mean of WRS in wn in the healthy persons and the dizzy patients.

Subject	Right** (**%)	Left (%)
Dizzy (affected ears)	51.4 ± 3.8	52.2 ± 3.5
Healthy	66.9 ± 9.3	67.5 ± 11.8
